# *MiR-27a* ameliorates inflammatory damage to the blood-spinal cord barrier after spinal cord ischemia: reperfusion injury in rats by downregulating TICAM-2 of the TLR_4_ signaling pathway

**DOI:** 10.1186/s12974-015-0246-3

**Published:** 2015-02-07

**Authors:** Xiao-Qian Li, Huang-Wei Lv, Zhi-Lin Wang, Wen-Fei Tan, Bo Fang, Hong Ma

**Affiliations:** Department of Anesthesiology, First Affiliated Hospital, China Medical University, Address: No. 155 Nanjing North Street, 110001 Shenyang, Liaoning China

**Keywords:** Blood-spinal cord barrier, MicroRNAs, MiR mimics, Anti-miR oligonucleotides, Toll-like receptor 4, Toll-like receptor adaptor molecule 2, Ischemia reperfusion injury

## Abstract

**Background:**

Spinal cord ischemia reperfusion (IR) injury causes inflammation and subsequently increases blood-spinal cord barrier leakage and Toll-like receptor 4 (TLR_4_) pathway activation. MicroRNAs (miRs) effectively regulate numerous target mRNAs during ischemia. However, their roles during IR injury are poorly understood. We investigated miRs involvement, particularly *miR-27a*, in TLR_4_ pathway-mediated inflammatory responses after IR.

**Method:**

We used a genomics approach to examine changed miRs of rats that had undergone 14 minutes of ischemia, followed by 24 or 72 hours of reperfusion. Quantitative RT-PCR was used to identify and confirm the miRs involved in regulating TLR_4_ pathway activation. We scanned miR databases for potential miR targets and confirmed these targets by quantitative RT-PCR. The miR mimic and anti-miR oligonucleotides (AMOs) were intrathecally injected at 12-hour intervals beginning three days before the ischemia. The effects of miRs on the TLR_4_ pathway and downstream cytokines were analyzed by PCR, western blotting, and ELISA. Double immunofluorescence staining was perfumed to determine the relationship between the targets and TLR_4_. Blood-spinal cord barrier (BSCB) permeability was examined using Evans blue (EB) dye.

**Results:**

A microarray analysis revealed that at 24 hours post-injury, three miRs were upregulated (>2.0 fold) and 15 miRs were downregulated (<0.5 fold), and at 72 hours, four miRs were upregulated and 14 were downregulated compared to their levels in sham-operated controls. We focused on *miR-27a*, which is predicted to contain sequences complementary to the 3'-untranslated region (UTR) of Toll-like receptor adaptor molecule 2 (TICAM-2). Double immunostaining indicated that TLR_4_ activation correlated with changes in TICAM-2 expression. Compared to the rats in the IR and negative control groups, intrathecal infusion of the *miR-27a* mimic attenuated IR-induced TLR_4_ activation and inflammatory damage to the BSCB, which was shown as decreased EB extravasation and lower levels of nuclear factor kappa-B (NF-κB) and lnterleukin (IL)-1β at 24 and 72 hours after reperfusion, whereas pretreatment with *miR-27a* AMO aggravated these injuries.

**Conclusions:**

We present the first evidence that miRs play an important role in spinal cord IR injury. We identified TICAM-2 as a novel target of *miR-27a. miR-27a* upregulation attenuates IR-induced inflammatory damage to the BSCB by negatively regulating TICAM-2 of the TLR_4_ signaling pathway and inhibiting the NF-κB/IL-1β pathway. These results provide new therapeutic targets for IR injury treatment.

## Background

Spinal cord ischemia reperfusion (IR) injuries have garnered much attention since 1986, when an IR injury was first reported, as they are associated with severe complications such as bladder, bowel, sexual dysfunction, and paraplegia [1]. Spinal cord IR injuries can induce a cascade of secondary events, such as neuronal or glial insults, that lead to further cell loss and behavioral impairments. These, in turn, are closely associated with inflammatory responses, including the release of cytokines, chemokines, and the recruitment of immune cells [2-4]. Numerous studies have shown that the major effectors involved in this inflammatory cascade activate the Toll-like receptors (TLRs), and the neuroprotective effect of inhibiting the TLR_4_ inflammatory pathway has been extensively studied by us and others [5-8].

We previously demonstrated that intrathecal antagonism of TLR_4_ in a rat model of IR, induced by transient occlusion of the aortic arch, could maintain the integrity of the blood-spinal cord barrier (BSCB) and attenuate secondary spinal cord injury by inhibiting proinflammatory cytokines, molecules downstream of the TLR_4_ pathway [6,7]. Contrary to previous investigations that showed that Myeloid differentiation factor_88__(_MyD_88)_ and MyD88 adaptor-like (Mal/TIRAP) adaptors functioned downstream of TLR_4_, the adapter TIR-containing adaptor molecule-2 (TICAM-2, also known as TRAM) was shown to resemble Mal/TIRAP and to physically bridge it with TICAM-1 to functionally transmit the TLR_4_ signal [9,10]. Therefore, TLR_4_ recruits two crucial adaptors, TIRAP and TICAM-2, which are connected to two effective adapters, MyD_88_ and TICAM-1, respectively, after activation [9].

MicroRNAs (miRs) are small, non-coding RNAs that are capable of specific binding to one or more target mRNAs, and effectively regulate their post-transcriptional expression in various tissues [11-13]. Studies have shown that several miRs can dramatically alter normal physiological processes and are involved in the pathogenesis of various diseases [14,15]. Recent studies showed that ischemia alters the expression of miRs in cardiac tissue, and antagomirs against miRs improved neovascularization and augmented functional recovery in a large animal model of cardiac IR injury, suggesting a role for miRs in the regulation of IR [16,17]. Furthermore, there is also increasing evidence for the involvement of miRs in traumatic spinal cord injury [18,19]. However, the role of miRs in IR is not well understood. Exploring the expression profiles of miRs altered after IR might reveal whether miR-dependent post-transcriptional gene regulation in TLR_4_-mediated inflammation determines the progression of, and recovery from, secondary damage to the spinal cord.

In this study, we first used miR arrays to determine the expression patterns of miRs in a rat model of spinal cord IR injury, and then identified their target mRNAs by searching the TargetScan, MicroCosm Targets (version 5), and microRNA.org databases. Among the miRs identified, we observed that *miR-27a* was one of the most dysregulated miRs, and further defined TICAM-2, a key regulator of the TLR_4_ pathway, as its target. Then, the effects of *miR-27a* were assessed in a rat model of IR by intrathecal pretreatment with an miR mimic and an anti-miR oligonucleotide (AMO) starting three days before ischemia. Our results demonstrated that increasing the expression of *miR-27a* attenuated IR-induced spinal cord injury by negatively modulating the TLR_4_ signaling regulator TICAM-2, which may be a new therapeutic target under neuroinflammatory conditions.

## Materials and methods

### Experimental animals

The experimental procedures were performed in accordance with the Guide for the Care and Use of Laboratory Animals (United States National Institutes of Health publication number 85–23, National Academy Press, Washington DC, revised 1996) The animals used in this study were male Sprague-Dawley rats (obtained from Animal center of China Medical University, Shenyang, China) weighing between 200 and 250 g. The rats were bred in standard cages with free access to food and water, and were housed separately after surgery at First Affiliated Hospital of China Medical University.

### Rat model of spinal cord ischemia reperfusion injury

To establish the spinal cord IR model, IR was induced by occluding the aortic arch for 14 minutes, as previously reported [6,7]. In brief, rats were anesthetized with an intraperitoneal injection of 4% sodium pentobarbital (Beyotime Biotechnology, Shanghai, China) at a dose of 50 mg/kg, and then the aortic arch was exposed through a cervicothoracic approach. Under direct visualization, the aortic arch was cross-clamped between the left common carotid artery and left subclavian artery. Occlusion was confirmed with a laser Doppler blood flow monitor (Moor Instruments, Axminster, Devon, United Kingdom). Ischemia, which is defined as a 90% decrease in the flow measured at the femoral artery{AU Query: If this definition originates from any official published guidelines, please provide a reference}, continued for 14 minutes, after which the clamps were removed and reperfusion was allowed to continue for either 24 or 72 hours. Sham-operated rats underwent the same procedure without aortic arch occlusion.

### MiR microarray analysis

To assess MiR expression in the spine, L_4–6_ segments of the spinal cord were harvested at 24 and 72 hours after reperfusion, frozen in liquid nitrogen, and stored at −80°C until use. Total RNA was isolated from the samples using TRIzol® reagent (Invitrogen, Carlsbad, California, United States) and the miRNeasy mini kit (Qiagen, West Sussex, United Kingdom) according to the manufacturers’ instructions. After measuring the quantity of RNA using a NanoDrop 1000 (Youpu Scientific Instrument Co., Ltd., Shanghai, China), the samples were labeled using the miRCURY™ Hy3™/Hy5™ Power labeling kit (Exiqon, Vedbaek, Denmark) and hybridized on a miRCURY™ LNA Array (version 18.0, Exiqon, Vedbaek, Denmark). After washing, the slides were scanned using an Axon GenePix 4000B microarray scanner (Axon Instruments, Foster City, California, United States). Scanned images were then imported into the GenePix Pro 6.0 program (Axon Instruments) for grid alignment and data extraction. Replicated miRs were averaged, and miRs with intensities of 50 or more in all samples were used to calculate a normalization factor. Expressed data were normalized by median normalization. After normalization, the miRs that were significantly differentially expressed were identified by Volcano Plot filtering. Finally, hierarchical clustering was performed to determine the differences in the miR expression profiles among the samples by using MEV software (version 4.6; TIGR, Microarray Software Suite 4, Boston, United States).

### Measurement of Evans blue extravasation

After 24 and 72 hours of reperfusion, Evans blue dye ((EB) 30 g/L; Sigma-Aldrich, Louis, United States) was intravenously injected (45 mg/kg) into the tail vein 60 minutes before the animals were euthanized. After adequate perfusion with saline under deep anesthesia, the L_4–6_ segments were removed, soaked in methanamide (Beyotime Biotechnology, Shanghai, China) for 24 hours at 60°C, and then centrifuged. EB content was measured as the absorbance of the supernatant at 632 nm on a microplate reader (BioTek, Winooski, Vermont, United States) and is reported as the amount of EB per wet tissue weight (μg/g). To measure the fluorescence, the tissue was fixed in 4% paraformaldehyde (Beyotime Biotechnology, Shanghai, China), sectioned (10 μm), sealed in a light-tight container, and frozen. EB staining was visualized using a BX-60 fluorescence microscope (Olympus, Melville, New York, United States) with a green filter.

### Quantification of miR expression

MiR expression was quantified by using an Applied Biosystems 7500 Real-Time PCR System (Foster City, California, United States) to verify regulation of the miR targets in the spinal segments of the IR and sham groups. Total RNA from the L_4–6_ segments of the spinal cords was extracted with TRIzol reagent and reverse transcribed to cDNA with the PrimeScript® miRNA cDNA synthesis kit (Perfect Real Time; TaKaRa, Dalian, China) according to the manufacturers’ instructions. PCR was then used to amplify *miR-27a* using SYBR Premix Ex TaqTM II (Perfect Real Time; TaKaRa, Tokyo, Japan) and *miR-27a*-specific primers (forward, 5′-ACACTCCAGCTGGGTTCACAGTGGCTAAG-3′ and reverse, 5′-TGGTGTCGTGGAGTCG-3′; RiboBio, Guangzhou, China) at 95°C for 10 seconds, followed by 40 cycles of 95°C for five seconds and 60°C for 20 seconds. The primers used to amplify U6 were 5′-CTCGCTTCGGCAGCACA-3′ (forward) and 5′-AACGCTTCACGAATTTGCGT-3′ (reverse). All reactions were performed in triplicate. The relative expression of *miR-27a* was normalized to U6. Data were analyzed by using the 2^−ΔΔCt^ method.

### Intrathecal pretreatment with a synthetic miR mimic and an anti-miR oligonucleotide

The method used to pretreat rats with a mimic and an AMO of *miRNA-27a* (GenBank number: [NR_031833.1]) and negative controls has been previously described [17]. For intrathecal infusion, a laminectomy was performed at the level of the thoracic vertebrae under pentobarbital anesthesia (Beyotime Biotechnology, Shanghai, China). A polyethylene catheter (PE10, Portex, Kent, United Kingdom, inside diameter (ID): 0.28 mm and outside diameter (OD): 0.61 mm) was passed caudally from T_9–12_, and 2 cm of the free end was left exposed in the upper thoracic region. We intrathecally infused 100 μL of a synthetic *miR-27a* mimic (*mimic-27a*), an AMO (*AMO-27a*), or the negative control (*NC-27a*, all at 50 mg/kg; Jima Inc., Shanghai, China) pretreated with Lipofectamine® 2000 (Invitrogen) continuously for three days before the surgical operation. The sequences of *mimic-27a*, *AMO-27a*, and *NC-27a* were 5′-UUCACAGUGGCUAAGUUCCGC-3′, 5′-GCGGAACTTAGCCACTGTGAA-3′, and 5′-AAGGCAAGCUGACCCUGAAGUU-3′, respectively. To analyze the specificity and efficacy of the *miR-27a* and *AMO-27a*, real-time PCR was performed as described above.

### Quantification of TLR_4_ and TICAM-2 mRNA

Quantitative real-time PCR was used to detect TLR_4_ and TICAM-2 mRNA as previously described [7]. Total RNA was extracted from L_4–6_ spinal cord tissue using TRIzol reagent according to the manufacturer’s instructions. PCR was performed as described previously using SYBR Green SuperMix-UDG on a Prism 7000 Sequence Detection System (Applied Biosystems) and the following primers: TLR_4_ (NM_0191178) forward, 5′-GGATGATGCCTCTCTTGCAT-3′ and reverse, 5′-TGATCCATGCATTGGTAGGTAA-3′; TICAM-2 (NM_021649) forward, 5′-GGGAATTCATAATGGGTATCGGGAAGTC-3′ and reverse, 5′-GGCTGCAGGTTATATGTTTCATCTCAGGC-3′; and GAPDH (glyceraldehyde-3-phosphate dehydrogenase, NM_023964) forward, 5′-AGAAGGCTGGGGCTCATTTG-3′ and reverse, 5′-AGGGGCCATCCACAGTCTTC-3′. Amplification was performed using the following cycling conditions: 50°C for two minutes (uracil-DNA glycosylase incubation), 95°C for 10 minutes, and 40 cycles of denaturation at 95°C for 15 seconds and annealing at 60°C for 30 seconds. All reactions were performed in triplicate. Gene expression was normalized to GAPDH (as an internal control). Data were analyzed by using the 2^-ΔΔCt^ method.

### Double immunofluorescence staining for TLR_4_ and TICAM-2

Double immunofluorescence staining was performed as described previously to explore the interaction between TLR_4_ and TICAM-2 after IR [6,7]. Briefly, 10-μm-thick sections were incubated with a primary rabbit anti-TLR_4_ antibody (1:800; Abcam, Cambridge, United States) and a goat anti-TICAM-2 antibody (1:100; Santa Cruz Biotechnology, Santa Cruz, California, United States) overnight at 4°C. After incubation with an Alexa 488-conjugated donkey anti-rabbit immunoglobulin G (IgG) antibody (1:500; Molecular Probes, Eugene, United States) and an Alexa 594-conjugated donkey anti-goat IgG antibody (1:500; Molecular Probes), each for two hours at room temperature, images were captured using a Leica TCS SP2 laser scanning spectral confocal microscope (Leica Microsystems, Buffalo Grove, Illinois, United States).

### Western blot analysis

The expression of TLR_4_, TICAM-2, and NF-κB p65 in spinal cord tissue was determined by western blot analyses. The rats’ spinal cords were homogenized, and total proteins were purified using tissue and nuclear protein extraction reagents according to the manufacturer’s instructions (KC-415 and KGP-150; KangChen, Shanghai, China). The antibodies against TLR_4_ (1:500; Abcam), TICAM-2 (1:500; Santa Cruz Biotechnology), and NF-κB p65 (1:500; Abcam) were used in this experiment, along with horseradish peroxidase-conjugated secondary antibodies (Bioss, Beijing, China). The scanned images were semi-quantitated using Quantity One software (Bio-Rad Laboratories, Milan, Italy).

### Measurement of lnterleukin -1β content using ELISA

The spinal cord was collected, homogenized, and then centrifuged. lnterleukin (IL)-1β content was determined with an ELISA kit (R&D Systems, Minneapolis, Minnesota, United States) according to the manufacturer’s instructions. Absorbance (A) was measured at 450 nm, and the IL-1β content of each sample was calculated based on a standard curve and was expressed in pictograms per milligram of total protein.

### Statistical analysis

All data were expressed as mean ± standard error of the mean (means ± SEM) and were analyzed with SPSS software (version 17.0; SPSS Inc., Chicago, Illinois, United States). All variables measured in this study were normally distributed, and the groups were compared with Student’s t-test or one-way analysis of variance (ANOVA), followed by Newman-Keuls *post-hoc* analysis. A *P* value less than 0.05 was considered to be statistically significant.

## Results

### Aberrant expression of miRs in the spinal cord after ischemia reperfusion

**Figure 1:**
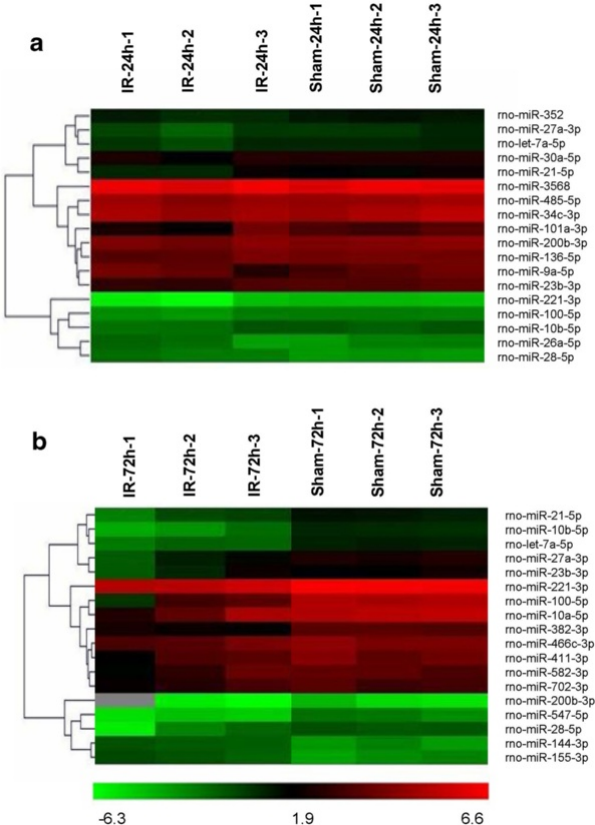
**Hierarchical cluster analyses of altered microRNAs (miRs) after spinal cord ischemia reperfusion (IR) injury. (a)** Differentially expressed miRs in the sham-operated and IR groups at 24 hours post-injury (n = three per group). **(b)** Differentially expressed miRs in the sham-operated and IR groups at 72 hours post-injury (n = three per group).Each row represents an miR and each column represents a sample. The color code shown at the bottom of the heat maps is linear, with green as the lowest and red as the highest. The miRs that were upregulated are shown in green to red, whereas the miRs that were downregulated are shown from red to green. Among all the significantly changed miRs, *miR-27a* was the most significantly downregulated in injured spinal cords at the abovementioned time points.

To determine the potential involvement of miRs in the spinal cord after IR, we used microarray analysis to determine miR levels in the spinal cord at 24 and 72 hours after reperfusion. The results showed that compared with the sham group, in the IR group, three miRs were upregulated (>2.0 fold) and 15 miRs were downregulated (<0.5 fold) at 24 hours after surgery (Figure 1a, Table 1), and four miRs were upregulated (>2.0 fold) and 14 miRs were downregulated (<0.5 fold) at 72 hours after surgery (Figure 1b, Table 2).

**Table 1:** **MicroRNAs (miRs) that were differentially expressed in the spinal cord of rats 24 hours after ischemia reperfusion (IR) injury compared with sham-operated rats (n = three per group)**

	**Average intensity of all probes (normalized)**		**Standard deviation**			
**miR**	**IR-24 hours**	**Sham-24 hours**	**IR-24 hours**	**Sham-24 hours**	**Fold change**	***P*** **value**
Upregulated						
rno-miR-3568	1.1817	0.3304	0.7156	0.3879	3.5766	0.0460
rno-miR-34c-3p	0.1437	0.6822	0.4047	0.8048	4.7460	0.1664
rno-miR-200b-3p	0.0201	0.1628	0.1434	0.7703	8.1081	0.1200
Downregulated						
rno-miR-352	2.0747	0.6335	0.2746	1.2941	0.3053	0.0667
rno-miR-27a-3p^a^	1.1155	0.4575	0.0362	0.7307	0.4101	0.0276
rno-let-7a-5p^a^	1.1155	0.5024	0.0362	1.1760	0.4504	0.1449
rno-miR-30a-5p	5.8525	1.8522	0.0380	1.2318	0.3164	0.0390
rno-miR-21-5p	3.7968	1.2200	0.1440	1.1157	0.3213	0.0383
rno-miR-485-5p	0.5746	0.3074	0.1272	0.2713	0.4954	0.0140
rno-miR-136-5p	26.647	10.0652	0.0403	1.0331	0.3777	0.0515
rno-miR-9a-5p	30.965	12.0872	0.1480	0.8460	0.3901	0.0433
rno-miR-101a-3p	20.289	10.5901	0.0886	0.4960	0.4919	0.0389
rno-miR-10b-5p^a^	17.175	7.4396	0.0651	0.8409	0.4331	0.0567
rno-miR-23b-3p^a^	10.737	5.2797	0.0318	0.8743	0.4918	0.1107
rno-miR-221-3p	0.5148	0.1355	0.1642	1.0404	0.3634	0.0162
rno-miR-26a-5p	1.9140	1.1414	0.1408	0.5557	0.4959	0.1241
rno-miR-100-5p	2.6130	1.1216	0.1617	0.6810	0.4293	0.0415
rno-miR-28-5p	0.0722	0.0286	0.2262	1.3509	0.3969	0.1642

Only the miRs with fold changes that were significantly different (upregulated >2.0 fold or downregulated <0.5 fold; *P* < 0.05) are shown.

##: ^a^miRs that were differentially expressed in the spinal cord at both 24 and 72 hours after IR.

**Table 2:** **MicroRNAs (miRs) that were differentially expressed in the spinal cord of rats 72 hours after ischemia reperfusion (IR) injury compared with that in the sham-operated rats (n = three per group)**

	**Average intensity of all probes (normalized)**		**Standard deviation**			
**miR**	**IR-72 hours**	**Sham-72 hours**	**IR-72 hours**	**Sham-72 hours**	**Fold change**	***P*** **value**
Upregulated						
rno-miR-144-3p	0.1945	0.8025	0.3912	0.2997	4.1246	0.0139
rno-miR-466c-3p	0.9230	1.5858	0.0982	0.2326	2.1179	0.0390
rno-miR-155-3p	0.1711	0.3344	0.0599	0.2500	4.1709	0.0201
rno-miR-200b-3p	0.0208	0.0627	0.1433	0.6803	3.1259	0.1591
Downregulated						
rno-miR-10a-5p	6.0399	3.4597	0.1390	0.0936	0.4957	0.0076
rno-miR-27a-3p^a^	1.1154	0.4575	0.0362	0.7307	0.3523	0.0276
rno-let-7a-5p^a^	0.7267	0.4315	1.5856	0.7777	0.4905	0.0206
rno-miR-382-3p	2.8900	1.3146	0.1144	0.1230	0.4548	0.0017
rno-miR-21-5p	3.7967	1.6388	0.1440	0.0352	0.4316	0.0024
rno-miR-411-3p	1.8435	1.1789	0.0852	0.0306	0.4939	0.0020
rno-miR-547-5p	0.3345	0.1405	0.2538	0.5311	0.4201	0.0410
rno-miR-702-3p	0.3098	0.10918	0.3002	0.1005	0.4102	0.0206
rno-miR-10b-5p^a^	17.174	11.0843	0.0650	0.0903	0.4645	0.0021
rno-miR-23b-3p^a^	10.736	6.6908	0.0318	0.0805	0.4623	0.0003
rno-miR-221-3p	0.5148	0.2680	0.1642	0.2330	0.4520	0.0152
rno-miR-582-3p	0.1262	0.0664	0.1170	0.2453	0.4526	0.0092
rno-miR-100-5p	2.6130	1.3189	0.1617	0.2232	0.4504	0.0121
rno-miR-28-5p	0.0722	0.0402	0.2261	0.0853	0.4557	0.0295

Only the miRs with fold changes that were significantly different (upregulated >2.0 fold, downregulated <0.5 fold; *P* < 0.05) are shown.

^a^##: miRs that were differentially expressed in the spinal cord at both 24 and 72 hours after IR.

**Figure 2:**
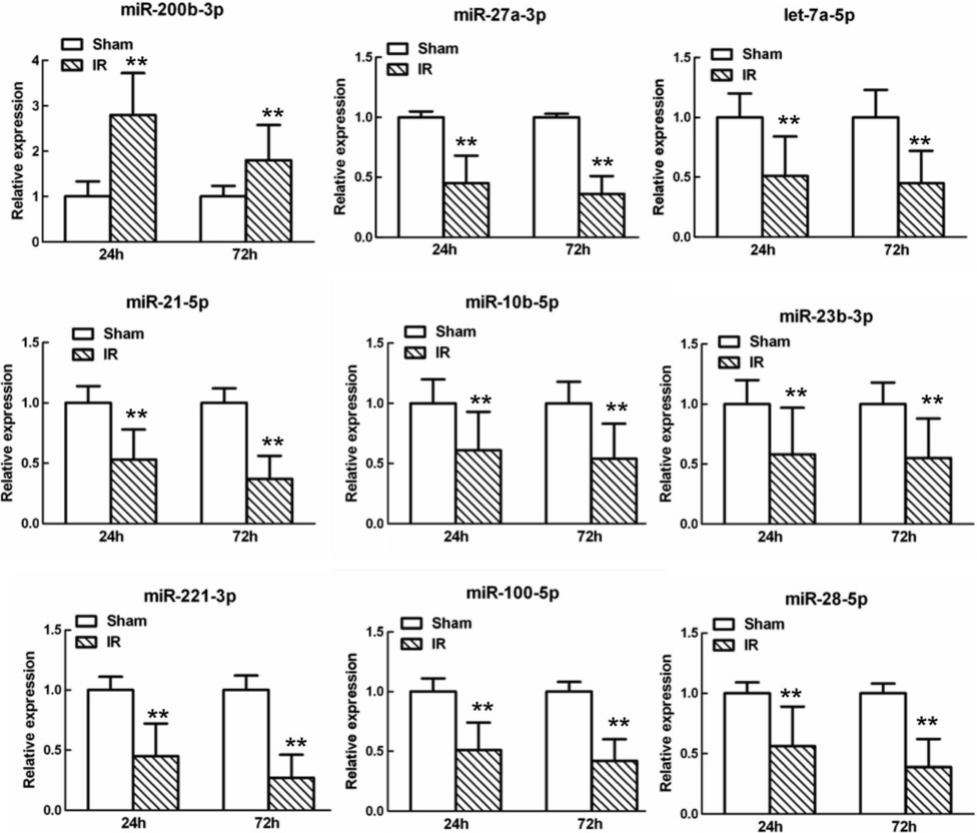
**Quantitative real-time polymerase chain reaction (qRT-PCR) analysis confirming the microarray analyses of the nine miRs that were abnormally expressed at both 24 and 72 hours after spinal cord ischemia reperfusion (IR).** Relative expression is the change in expression compared to the sham-operated group at either 24 or 72 hours after reperfusion. Data are expressed as mean ± SEM. **P <0.05 versus the sham group.

**Figure 3:**
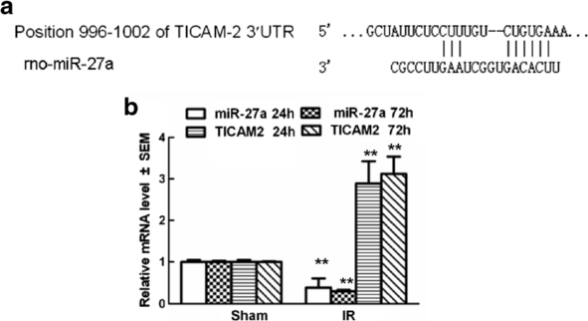
**A putative target site of** ***miR-27a*** **located in the 3′-UTR of TICAM-2 mRNA was predicted by bioinformatics analysis. (a)** The predicted *miR-27a* binding site in the 3′-untranslated region (UTR) of TICAM-2 in rats. **(b)** PCR analysis confirming TICAM-2 mRNA was abnormally expressed at both 24 and 72 hours after spinal cord ischemia reperfusion (IR). The profiles of TICAM-2 were expressed in an opposite manner as those of *miR-27a*. Relative expression is the change in expression compared to the sham-operated group at either 24 or 72 hours after reperfusion. Data are expressed as mean ± SEM. **P <0.05 versus the sham group. Results shown are representative data from three separate experiments.

Nine of the miRs (*miR-200b-3p*, *miR-27a-3p*, *let-7a-5p*, *miR-21-5p*, *miR-10b-5p*, *miR-23b-3p*, *miR-221-3p*, *miR-100-5p*, and *miR-28-5p*) were differently expressed at both 24 and 72 hours after reperfusion. Among them, *miR-27a-3p*, *let-7a-5p*, *miR-10b-5p*, and *miR-23b-3p* have been shown to function as regulators of spinal cord development and remodeling, and have been implicated in diseases of the spinal cord [20,21]. Their abnormal expression was confirmed by quantitative real-time polymerase chain reaction (qRT-PCR) (Figure 2). The results showed that *miR-27a* was expressed at significantly low levels after IR and continuously decreased with time (*P* < 0.05, versus the sham group). In addition, searching the TargetScanHuman 6.2 and MicroCosm Targets version 5databases revealed a perfect match to a *miR-27a* binding site in the 3′-UTR of the TICAM-2 gene (Figure 3a), which was confirmed by quantitative real-timeq RT-PCR (Figure 3b). Therefore, we hypothesized that downregulation of *miR-27a* might play a role in mediating TLR_4_-mediated secondary inflammatory damage after IR by up-regulating the TICAM-2 transcript.

### Intrathecal pretreatment with an *miR-27a* mimic and anti-miR oligonucleotides successfully regulated TICAM-2 expression after ischemia reperfusion

**Figure 4:**
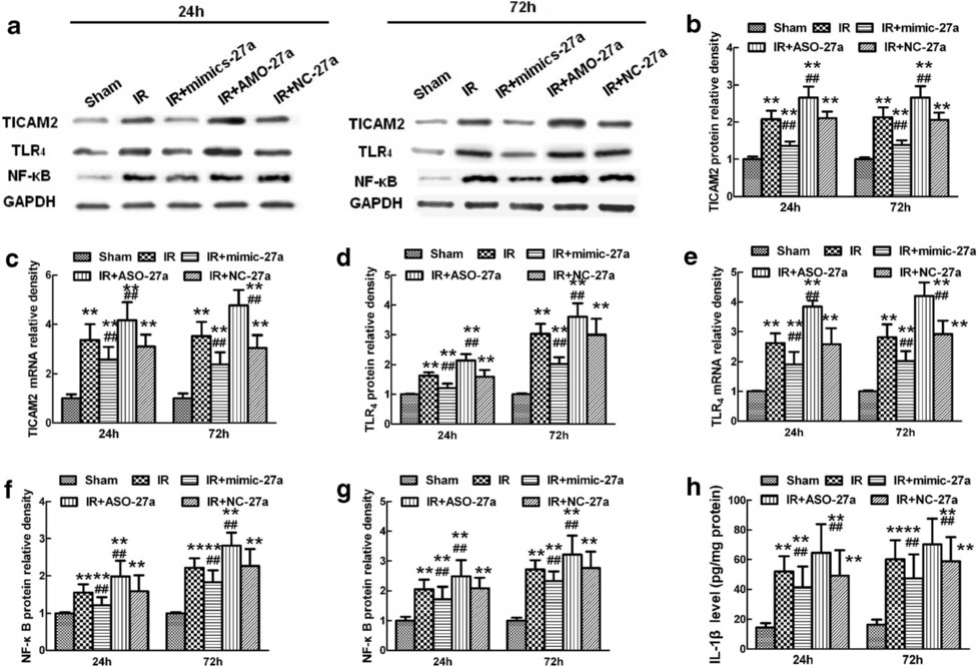
**Effects of intrathecal injection of** ***mimic-27a*** **and** ***AMO-27a*** **on the expression of TICAM-2 and the TLR**_**4**_ **/NF-** **κB/IL-1β pathway in rats after spinal cord ischemia reperfusion (IR). (a)** Representative immunoblots were probed with antibodies against TICAM-2, TLR_4_, and nuclear NF-κB p65, and an antibody against GAPDH served as a loading control. **(b–g)**Quantification of the densities of the *TICAM-2* **(b)**, *TLR*_*4*_ **(d)**, and *NF-κB p65* **(f)** bands in nuclear extracts under different conditions. Protein expression is presented in relative units. Real-time PCR analyses of *TICAM-2* **(c)**, *TLR*_*4*_ **(e)**, and *NF-*κ*B p65* **(g)** were performed in duplicate and normalized to *GAPDH* mRNA levels. **(h)** Quantification of IL-1β production in the spinal cord at 24 and 72 hours after IR injury, as assessed by ELISA. IR caused significant increases in TICAM-2 expression, and intrathecal injection of *mimic-27a* and *AMO-27a* altered TICAM-2 expression after IR. In accordance with the decreased TICAM-2 expression, increased *miR-27a* levels induced by intrathecal injection of *mimic-27a* also prevented the increases in TLR_4_ and nuclear NF-κB p65 expression, as well as the increased IL-1β levels, whereas suppression of *miR-27a* by treatment with *AMO-27a* reversed the above changes, indicating that the TLR_4_/NF-κB/IL-1β pathway is involved in the *miR-27a*-mediated regulation of inflammation in the spinal cord through *TICAM-2* targeting. All data are presented as mean ± SEM. **P <0.05 versus the sham group; ^##^ *P* <0.05 versus the IR group.

To explore the effects of a synthetic *miR-27a* mimic (*mimic-27a*), *miR-27a* AMO (*AMO-27a*), and a negative control mRNA (*NC-27a*) on TICAM-2 expression in the spinal cord after IR, we intrathecally injected *mimic-27a*, *AMO-27a*, and *NC-27a* for three days before ischemia, and then examined both the mRNA and protein expression of TICAM-2 by RT-PCR and western blotting, respectively. As shown in Figure 4a, b, c, compared with the negative control (*NC-27a*), intrathecal injection with *mimic-27a* significantly prevented the IR-induced increases in TICAM-2 mRNA and protein expression at 24 and 72 hours after reperfusion, whereas injection with *AMO-27a* reversed these effects (*P* < 0.05 versus the *NC-27a* group). Compared to the IR group, intrathecal injection of *NC-27a* before ischemia induced no obvious differences at 24 and 72 hours after reperfusion (*P* > 0.05 versus the IR group).

### Expression and colocalization of TLR_4_ and TICAM-2 after ischemia reperfusion

**Figure 5:**
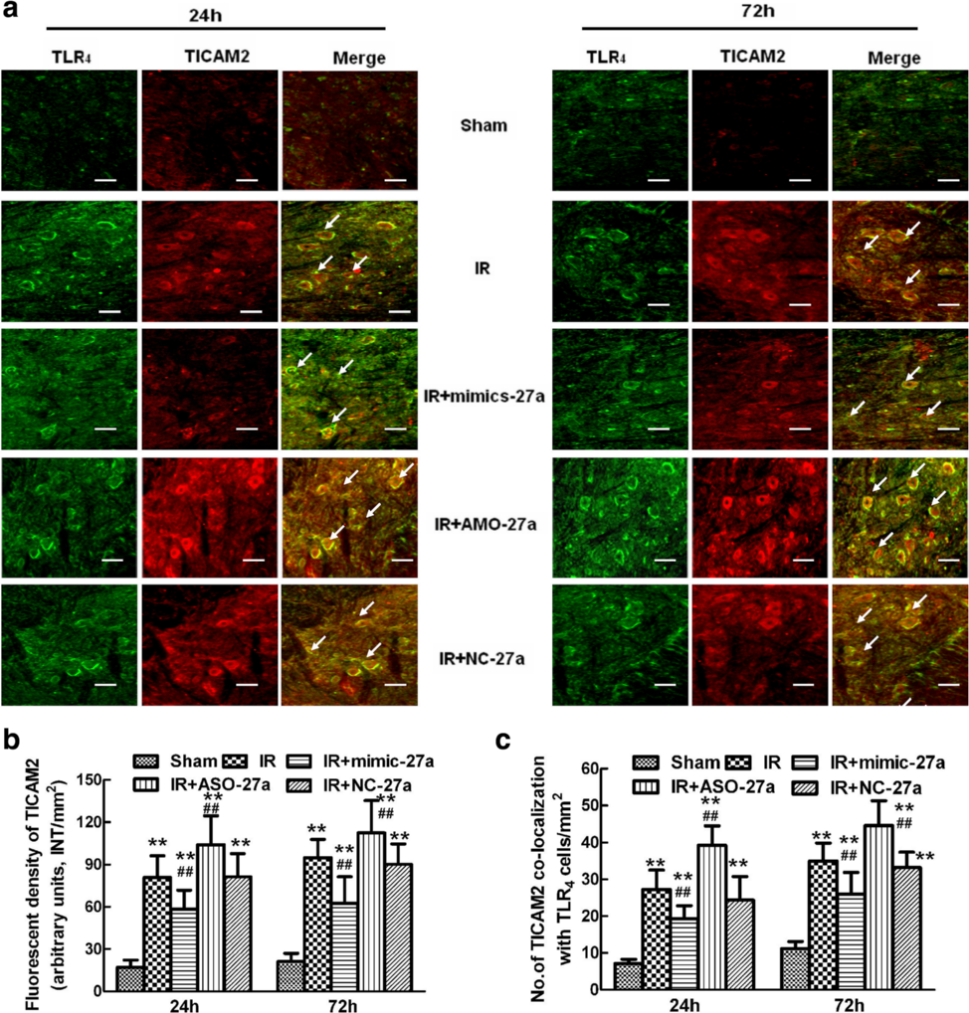
**Effects of intrathecal injection of** ***mimic-27a*** **and** ***AMO-27a*** **on the expression and colocalization of TLR**_**4**_ **and TICAM-2** ***in vivo*** **after spinal cord ischemia reperfusion (IR). (a)** Representative micrographs showing the colocalization of TLR_4_ (green) and TICAM-2 (Red) at 24 and 72 hours after IR injury. Arrows show their co-localization. Scale bars = 100 μm. **(b)** Quantification of TICAM-2 immunoreactivity is presented as the average fluorescence intensity (FI) of three independent experiments. **(c)** Histogram for the quantification of co-localized cells (cells with yellow signals). Double fluorescence immunohistochemistry showing that membrane-bound TLR_4_ has an expression profile that is similar to that of TICAM-2 in the cytoplasm of neurons and glial cells from injured regions of the spinal cord at 24 and 72 hours after surgery. Increasing the levels of *miR-27a* by intrathecal pretreatment with *mimic-27a* significantly decreased TICAM-2 immunoreactivity after IR and the number of TICAM-2-TLR_4_ positive cells, whereas these effects were reversed by intrathecal injection of *AMO-27a*. All data are presented as mean ± SEM. **P <0.01 versus the sham group; ^##^ *P* <0.05 versus the IR group.

TICAM-2 is a cytoplasmic protein that is known to be associated with TLR_4_ signaling. The relationship between TICAM-2 and TLR_4_ expression in the spinal cord 24 and 72 hours after reperfusion was examined by double immunofluorescence staining. As shown in Figure 5a, the level of membrane-bound TLR_4_ immunostaining was similar to that of cytoplasmic TICAM-2 immunostaining in the neurons and glial cells of injured spinal cords, but not in sham-operated ones, which confirmed that TLR_4_ was indeed upregulated when TICAM-2 was upregulated during IR. Quantification of TICAM-2 expression is shown in Figure 5b (*P* < 0.05 versus the IR group).

Conversely, compared to the IR group, increased *miR-27a* levels by pretreatment with *mimic-27a* reduced TICAM-2 immunoreactivity and the number of double-labeled cells in lumbar spinal cords during the 72-hour follow-up period. In contrast, much stronger TICAM-2 immunoreactivity and more double-labeled cells were detected in the spinal cords of rats pretreated with *AMO-27a* (*P* < 0.05 versus the IR group). No significant difference was observed in operated rats with or without *NC-27a* treatment at either 24 or 72 hours (*P* > 0.05 versus the IR group). Quantification of the double-labeled cells is shown in Figure 5c.

### Intrathecal pretreatment with *mimic-27a* attenuated blood-spinal cord barrier (BSCB) leakage after ischemia reperfusion 

**Figure 6:**
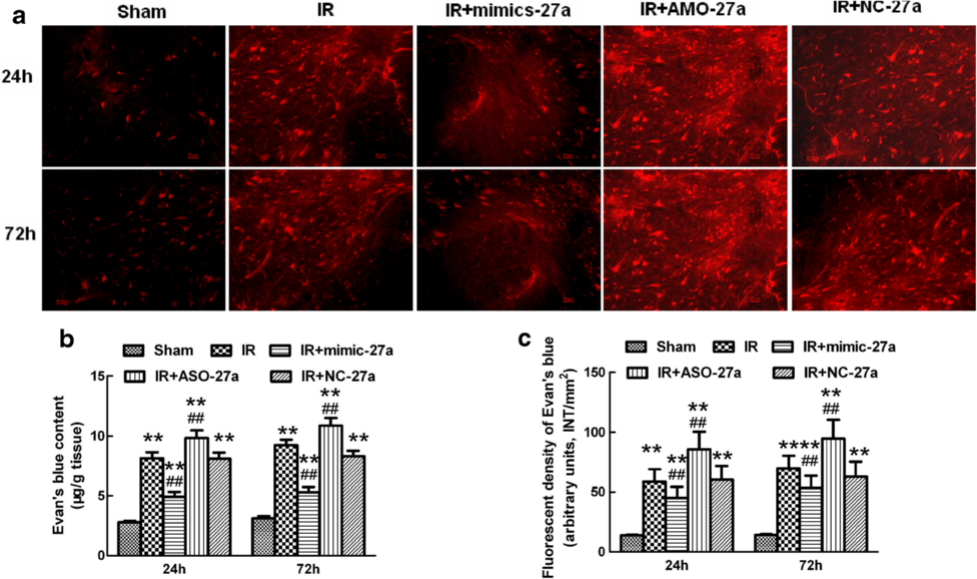
**Effects of intrathecal injection of mimic-27a and AMO-27a on blood-spinal cord barrier (BSCB) integrity after ischemia reperfusion (IR). (a)** Effects of intrathecal injection of *mimic-27a* and *AMO-27a* on BSCB permeability measured by Evans blue (EB) extravasation. Almost no red fluoresce was seen in the spinal cord of the sham group at 24 and 72 hours after IR. Compared with the IR group, EB extravasation (red) was significantly lower in rats who received an intrathecal injection of *mimic-27a*. Conversely, at 24 hours after injury, much more red fluorescence, especially in the gray matter, was seen in rats who received an intrathecal injection of *AMO-27a,* which was even stronger at 72 hours post-injury. **(b)** The EB content of the spinal cord (μg/g). **(c)** Quantification of EB fluorescence density (INT/mm^2^) . Original magnification, 100×; Scale bar = 50 μm. **P <0.05 versus the sham group; ^##^ *P* <0.05 versus the IR group.

EB content and fluorescent dye were used to assess IR-induced BSCB disruption as described previously [6,7,22]. BSCB permeability was visualized by EB extravasation. As shown in Figure 6a, compared with the sham group, IR caused a marked increase in EB extravasation at 24 and 72 hours after reperfusion (*P* < 0.05 versus the sham group). Intrathecal infusion of *mimic-27a* attenuated BSCB dysfunction, as evidenced by the decreased EB extravasation and fluorescent dye content at 24 and 72 hours after reperfusion, whereas intrathecal infusion of *AMO-27a* synergistically exacerbated BSCB leakage (*P* < 0.05 versus the IR group). There were no detectable differences in EB extravasation between the IR group and the group pretreated with *NC-27a* at all time points (*P* > 0.05). The quantification of EB content in the spinal cord shown in Figure 6b and the fluorescent densities shown in Figure 6c confirmed the above results.

### Intrathecal pretreatment with *mimic-27a* inhibited TLR_4_/NF-κB/IL-1β activation after ischemia reperfusion

To further confirm that *miR-27a* is involved in TLR_4_ activation by regulating the expression of TICAM-2 after IR, we examined the mRNA and protein levels of TLR_4_, NF-κB, and the downstream inflammatory cytokine IL-1β by ELISA. In agreement with the double immunofluorescence TICAM-2 and TLR_4_ staining, intrathecal pretreatment with *mimic-27a* reduced the IR-induced TLR_4_ and NF-κB mRNA and protein levels at 24 and 72 hours after reperfusion (Figure 4a, d, e, f; *P* < 0.05 versus the IR group). In contrast, intrathecal infusion of *AMO-27a* increased TLR_4_ and NF-κB mRNA and protein levels (*P* < 0.05 versus the IR group) at all time points. Compared to the IR group, there were no detectable differences in TLR_4_ and NF-κB expression when animals were pretreated with *NC-27a* (*P* > 0.05). Furthermore, levels of the downstream inflammatory cytokine IL-1β also increased with TLR_4_ and NF-κB increasing (Figure 4h; *P* < 0.05 versus the IR group). These results are consistent with the hypothesis that the IR-mediated reduction in *miR-27a* down-regulates TICAM-2 expression, which in turn influences TLR_4_ signaling and the activation of downstream inflammatory cytokines.

## Discussion

The BSCB is a physical and biochemical barrier between the circulation and the spinal cord that plays an important role in the regulation of spinal cord homeostasis. Disruption of BSCB integrity after IR injury has been reported to trigger alterations in the spinal microenvironment, allowing penetration of both inflammatory cytokines and immune cells into the spinal cord, which determines the prognosis of patients with IR injury [6,7,22]. Accumulating evidence indicates that some of the major effectors in this inflammatory damage to BSCB are engagement with toll-like receptors (the TLRs) signal pathway [5-8,23]. Recently, many studies have shown that individual miRs, endogenous, non-coding, single-stranded, small RNAs that are widespread in eukaryotic organisms, are capable of affecting numerous target mRNAs and effectively regulating their gene expression [8,15,18,19]. Altering the expression of miRs greatly affects the pathogenesis of IR and its functional outcome [16,24]. Therefore, in this present study, we provide evidences that miRs attenuate BSCB permeability, and our findings expand the understanding of the molecular mechanisms underlying TLR_4_-mediated inflammatory responses. To the best of our knowledge, this is the first study to document a *miR-27a* target related to the TLR_4_ pathway in spinal cord IR injury.

MiRs are a class of sophisticated gene expression regulators with the unique ability to prevent the translation of and/or degrade their corresponding target mRNAs by inhibiting translation and promoting cleavage, respectively. MiRs act as key regulators in a wide variety of biological processes, including cell proliferation, differentiation, apoptosis, and organ development, and have also been implicated in inflammatory diseases [25,26]. Previous studies showed that some miRs may regulate genes associated with TLR signaling pathways; the mRNA expression of five adaptor and interacting proteins (CD14, HSPA1a, Pglyrp-1, MD-1, and TICAM-2) were significantly upregulated following traumatic brain injury (TBI) [8,23]. In this study, using a miRs microarray screening approach, we found that *miR-27a* expression was significantly altered at both 24 h and 72 hours after IR, and such results were as confirmed by qRT-qPCR (Figure 2).

In animals, recognition of miR response elements only requires a continuous 6six-base pair ‘“seed match”’ near the 3′-UTR of its target mRNA [27]. Given this, many target genes of *miR-27a* have been identified, some of which were showed to participated in innate immune response and inflammatory responses [28-31]. Whether *miR-27a* specifically target TICAM-2 *in vivo* remainsed to be tested. It hasis recently been shown that pretreatment with miR mimics and AMOs was is one of the most common and useful methods to regulate the expression levels of miRs [16,17,28,29]. He *et al*. reported that injecting miR mimics 48 hours before IR greatly downregulated the levels of apoptosis-related genes, whereas injecting the corresponding AMOs upregulated their expression [17]. Our *in vivo* data, shown in Figure 4a, b, c, are in close accordance with previous studies, showing that compensating for decreased *miR-27a* levels by continuous intrathecal injection of *mimic-27a* into the subarachnoid space of IR model rats, reduced the mRNA and protein expression of TICAM-2 by inhibiting the translation and/or promoting the degradation of its mRNA. Continuous intrathecal injection of *AMO-27a* before ischemia clearly abrogated reversed such changes. However, no obvious changes were detected when model animals were pretreated with *NC-27a*, suggesting that *miR-27a* directly modulates TICAM-2 expression i*n vivo*, and that modulation of *miR-27a* is particularly important in the pathophysiology of IR.

TICAM-2 is a cytoplasmic protein that structurally resembles the MAL/TIRAP adaptor that links TLR_4_ and MyD_88_ and functionally transmits TLR_4_ signaling to TICAM-1 [9,32]. Given the bridging action between TLR_4_ and TRIF, TICAM-2 coordinates the inflammatory response to a pathogen challenge [33]. Thus, it is easy to postulate that IR-induced aberrant TICAM-2 expression might be closely associated with TLR_4_ activation, subsequent NF-κB relocation to the nucleus, and the release of downstream proinflammatory cytokines [9,32,34]. Consistent with this, here, we observe that TLR_4_ expression is also significantly upregulated when TICAM-2 expression increased and *miR-27a* decreased at both 24 and 72 hours after IR (Figure 4a, b, c, d, e). In addition, the double immunofluorescence staining in Figure 5 shows that the signal for TLR_4_, a membrane-bound receptor, is greatly upregulated coincident along with increased cytoplasmic staining of TICAM-2 in neuronal and glial cells of ischemic spinal cords. Therefore, treatments that decrease TICAM-2 and inhibit the inflammatory stimulation of TLR_4_ might be a novel intervention for IR [9,32,33,35]. We then identifiedy the mechanism underlying miR-dependent regulation of TICAM-2 expression. Intrathecal injection of *mimic-27a* prevented the increase in TICAM-2 immunoreactivity and the number of double-labeled cells, whereas intrathecal injection of *AMO-27a* reversed these effects. In accordance with these observed effects of mimic-27a and *AMO-27a* on TICAM-2, we observed similar expression profiles for TLR_4_, NF-κB, and the proinflammatory cytokine IL-1β at 24 and 72 hours post-injury, providing direct evidence that IR-induced TLR_4_ activation is significantly influenced by the expression of TICAM-2.

Proinflammatory cytokines are important molecules in the immune system that have been implicated in alterations of BSCB integrity [6,7,36,37]. Breakdown of the BSCB allows exogenous pathogens and circulating immune cells to enter the spinal cord, which has numerous consequences, including neuronal loss, central sensitization, and glial remodeling [22,36]. Inhibiting inflammatory damage to the BSCB has been postulated as thea key to protection in IR. Our present study provides clear evidence for the protective effects of specifically increasing *miR-27a* expression. An Iinjection of *mimic-27a* attenuated BSCB dysfunction, which was manifested as reduced fluorescent dye and EB extravasation, and was suppressed by the injection of *AMO-27a*; this finding indicatesd that *miR-27a* regulates inflammatory damage to the BSCB by targeting the TICAM-2 mRNA and the TLR_4_/NF-κB/IL-1β pathway.

Significantly, one major character of miRs is that a single miR is capable of regulating the expression of many target genes, whereas a target gene can also be regulated by several miRs [11,13,26]. Thus, it is very possible to gain different or even contradictory expressions when exploring the same miR in differently experimental conditions [20,28,38,39]. For example, in the recsent study of Young *et al*. showed the opposite role of *miR-27a* in the regulation of vascular leaking by targeting vascular endothelium (VE)-cadherin in the endothelium [38]. On the other hand, some studies showed an upregulation of *miR-27a* for systemic inflammation, instead of the down-regulation as described in this study in the event of systemic inflammation [28,39]. These disagreements might be caused by the different observation time points, which are consistent with the descriptions of that no changes being observed in *miR-27a* expression for at 6 hours, instead of downregulation for at 24 hours after lipopolysaccharide (LPS) treatments [28]. Moreover, the net effects of miRs observed in *in vivo* experiments were also influenced by the complicated internal environment [20,40]. Luxenhofer G *et al*. emphasized the importance of miRs in regulating the molecular network specifying the generation of neuronal diversity in the developing chick spinal cord [40]. Ziu M *et al*. also showed significantly different expression levels of miRs in prolonged compression injury compared to those in short compression injury [20]. Furthermore, different expressions of miRs were reported in different regions of the injured spinal cord, even under the same experimental conditions [20]. Thus, aseptically inflammatory responses during spinal cord IR injury could not be exactly equivalent to directly proinflammatory stimuli with LPS *in vitro* as well as the results obtained from the lung tissue. Further *in vitro* and *in vivo* studies still need to be conducted to identify the correlation between *miR-27a* and the corresponding target genes in the mode of inflammatory or anti-inflammatory actions to better elucidate the mechanism and provide potential therapeutic targets for IR.

Taken together, we identify TICAM-2 as a novel target of *miR-27a* and show that downregulation of *miR-27a* promotes IR-induced inflammatory damage to the BSCB by facilitating activation of the TLR_4_/NF-κB/IL-1β signaling pathway.

## Abbreviations

AMO: Anti-miRNA oligonucleotides
BSA: Bovine serum albumin
BSCB: Blood-spinal cord barrier
EB: Evans blue
GAPDH: Glyceraldehyde-3-phosphate dehydrogenase
IR: Ischemia-reperfusion
ID: Inside diameter
IgG: Immunoglobulin G
IL: Interleukin
INT: Intensity
IRF3: Interferon regulatory factor 3
LPS: Lipopolysaccharide
miRs: microRNAs
MyD_88_: Myeloid differentiation factor _88_
Mal/TIRAP: MyD88 adaptor-like
NC: Negative control
NF-κB: Nuclear factor kappa-B
OD: Outside diameter
qRT-PCR: Quantitative real-time polymerase chain reaction
SEM: Standard error of the mean
TBI: Traumatic brain injury
TLR_4_: Toll-like receptor 4
TICAM-2: Toll-like receptor adaptor molecule 2
TRIF: TIR domain-containing adaptor-inducing IFN-β
UTR: Untranslated region
